# The Truth Shall Make Us Free

**Published:** 1896-08

**Authors:** J. H. Allen

**Affiliations:** Logansport, Ind.


### "THE TRUTH SHALL MAKE US FREE."

(FIFTH ARTICLE.)

J. H. ALLEN, M. D., LOGANSPORT, IND.

Truth unfolds the curtains of the night, and the shadows that were cast by ignorance and agnosticism over the true relationship of man's existence toward his Creator, and all true biological law, are dissolved into nothingness, by that blazing star of truth that crowned the forehead of that morn in which Hahnemann discovered the grace-given principle Similia, by whose powers the love of God could be made more manifest to the sick and pain-suffering one through us as physicians. By the application of the truth in science, for offended law can only seek reconciliation through law, and this reconciliation or restored harmony in the sick one comes only when law and grace have kissed each other through Similia. God has given to the human soul the inherent power under certain conditions to perceive and comprehend the fixed laws of Nature. What these conditions are we may never know, but they do exist. We find one perceiving the law of sound, another of color, of light, of harmony, of chemical action, of Similia. The cure of disease through law is so silent, so symmetrical, so harmonious, that we cannot believe it is the workings of law; we look for signs and symbols but we find but silence, action, and invisible change; only through the study of phenomena can we see its action. The working of all law is consummate wisdom; in it we have the key of life, of power, and of harmony; indeed it is the source of applied power or the hand of God in action. So that power applied through it is true harmonics, but if applied out of law it is perverted power, and perverted power in therapeutics is disease-producing in its action, where, on the other hand, if the power is applied through the law of Similia it is health-producing, health-restoring, magnifying all the functions of the organism. No! no! cries one that knows not all the truth, Homœopathy does not look like that to me. Ah, my dear worker in the field of Homœopathy, you have not pulled down all the wall, so as to let in all the light you can; climb higher and higher and stand face to face with the Master Hahnemann. Then look down upon the climbers and the workers in the fields. Not all may be able to see the truth as we do, yet it is hoped they will some day. Aspire to it, dear readers, and some time the "truth shall make you free" in the broadest acceptation of the word. Oh! how we all admire Hahnemann for his loftiness. He stopped far up the slope and the truth made him a king and the angel side of our human love and ambition is ever climbing up to be crowned by him. He was a master, and yet in memory methinks he stands with many others in science, in the noonday glare of that great white light of truth that lighteth up the world, inspiring us to higher motives and to higher things, so that we cannot do better than to climb, calling unto him from the heights. And soon the revelation will come and we are made partakers of that freedom that only the truth reveals. Thus are we led into the presence of the majesty and power of potency. Thus we are ushered into a larger life in science from a lesser life. And having heretofore only a human conception of the law of cure, now we have a divine conception, because we are now willing to be guided by the precepts and principles of law. Thus we are lifted up by and through the principles that govern our science.

All nature is but an expression of divine love; we see it in its beauty in the crystal, a higher form of it in vegetable, and a still higher in the animal, and crowned in all its glory in man, who is the epitome of all organized life. Indeed, love is the great moving principle of all harmonics, whether it be grace or law. So Homœopathy throws off the case-hardened wrappings of the ages, and reveals through Similia the great, deep, sacred love of infinity that is ever falling as a benediction upon our heads. Then let us scrape off the barnacles of empiricism and unhomœopathic methods lest they sink the ship, and meet the onslaughts of the enemy by ever presenting before them the mirror of truth, so that they may have a true reflection of their *skeleton* in *armor*. Let us clothe ourselves with the whole armor of truth as it is taught in pure Hahnemannian Homœopathy, wearing for a breast-plate Similia and engraven on our shield of faith Simplex, Similia, Minimum; studying the phenomena of disease and not the nomenclature; studying each case not as a disease by name, but as a disturbance of the life-force, as a new conception, an entirely different manifestation of perverted biological law; a disturbance of that peculiar complex relationship between body, mind, and spirit; that relationship that is so intimate—Dynamic, Triuneus, Omnipresentius, Animalis, Humanus, Deus—with its potens of body earthy, potens of mind intellectual, potens of spirit divinity; maintaining a relationship to mother earth in its earthy body, manifesting its relationship to wisdom through that great intermedium, the mind, manifesting its relationship to its Creator through the spirit. Thus when we say "ashes to ashes, dust to dust," we mean that he cannot take from earth that which belongs to earth, nor that from mind that belongs to mind—which is but the potens of the soul or spirit, which is of God. But it devolves on us as physicians to maintain that true relationship or harmony by a true knowledge of physical law, so as to be able to suggest to him his proper relationship to that law and a true knowledge of biological law; so as to teach him the internal workings of biological dynamics and a true knowledge of the relationship between mind, body, and spirit, so as to not prevent that true, happy, and harmonic relationship. From perverted mind we have perverted life-force, or *vice versa,* which may be followed by structural or functional changes in the organism even to a pathological condition. A spiritual or a mental aberration may produce a change in the physical organism, which, being classified under some special name in our nomenclature, at once ushers us into the domain of antipathic medicine, and robs disease of its dynamic nature, clothes it with the unscientific garb of materialism, and takes away all those wonderful and infinite phenomena of disease that give us so many and infinite expressions of the disturbed life-force, and there remains nothing except a pathological grouping.

